# Peritonitis secondary to traumatic duodenal laceration in the presence of a large pancreatic pseudocyst: a case report

**DOI:** 10.1186/1752-1947-5-528

**Published:** 2011-10-26

**Authors:** Vanessa RE Tuboku-Metzger, Marlon M Seenath, Lam Chin Tan

**Affiliations:** 1 University Hospital Coventry and Warwickshire, Clifford Bridge Road, Coventry, CV2 2DX, UK

## Abstract

**Introduction:**

A pancreatic pseudocyst is a common sequela of severe acute pancreatitis. Commonly, it presents with abdominal pain and a mass in the epigastrium several weeks after the acute episode and can be managed conservatively, endoscopically or surgically. We report a patient with a pancreatic pseudocyst awaiting endoscopic therapy who developed a life-threatening complication following a rather innocuous trauma to the abdomen.

**Case presentation:**

A 23-year-old Asian male student presented as an emergency with an acute abdomen a week after a minor trauma to his upper abdomen. The injury occurred when he was innocently punched in the abdomen by a friend. He experienced only moderate discomfort briefly at the time. His past medical history included coeliac disease and an admission four months previously with severe acute pancreatitis. He was hospitalized for 15 days; his pancreatitis was thought to be due to alcohol binge drinking on weekends. Ultrasound scanning showed no evidence of gallstone disease. Five days after the trauma, he became anorexic, lethargic and feverish and started vomiting bilious content. Seven days post-trauma, he presented to our emergency department with severe abdominal pain. An emergency laparotomy was performed where a transverse linear duodenal laceration was found at the junction of the first and second part of his duodenum, with generalized peritonitis. His stomach and duodenum were stretched over a large pancreatic pseudocyst posterior to his stomach. It was postulated that an incomplete duodenal injury (possibly a serosal tear) occurred following the initial minor trauma, which was followed by local tissue necrosis at the injury site resulting in a delayed presentation of generalized peritonitis.

**Conclusion:**

This is the first reported case of a traumatic duodenal laceration following minor blunt trauma in the presence of a large pancreatic pseudocyst. Minor blunt abdominal trauma in a normal healthy adult would not be expected to result in a significant duodenal injury. In the presence of a large pseudocyst, however, the stretching of the duodenum over the pseudocyst had probably predisposed the duodenum to this injury. Patients awaiting therapeutic interventions for their pancreatic pseudocysts should be warned about this unusual but life-threatening risk following minor blunt abdominal trauma.

## Introduction

A pancreatic pseudocyst can occur secondary to pancreatic duct disruption, such as that which occurs during an episode of severe acute pancreatitis. Pathologically, it is a localized collection of pancreatic secretions lacking an epithelial lining [1]. Complications of pancreatic pseudocysts include infection, hemorrhage and rupture. The drainage of pseudocysts, either with surgery or radiology, is therefore employed to prevent these complications [1].

Duodenal laceration may occur as a result of blunt or penetrating trauma, with injuries occurring as a result of blunt trauma being less common [2]. A considerable force is necessary for blunt abdominal trauma to result in a duodenal injury [2]. A review of patients admitted to eight trauma centers over a five-year period demonstrated that death following a blunt duodenal injury was rare, with deaths usually due to an associated hepatic or vascular injury [3]. Blunt duodenal injuries can, however, be a source of morbidity. In the aforementioned review, duodenal dehiscence, fistulas and intra-abdominal abscesses were the main causes of morbidity [3]. Most duodenal injuries can be managed by surgical repair [3]. Non-operative management of duodenal perforations with intravenous antibiotics and intravenous fluids has been described [4]. Patients most suitable for conservative management are those who are not septic and displaying only mild symptoms and signs. These patients will usually have duodenal perforations that have or will spontaneously seal off [4].

A review of the current literature, searching for the terms 'pancreatic', 'pseudocyst', 'blunt', 'trauma', 'gastrointestinal' and 'injuries' found three articles using the Medline and Embase databases. A retrospective study of 203 children who suffered intra-abdominal trauma following blunt injuries showed that 12 children had sustained gastrointestinal perforations, but none were in the presence of a pancreatic pseudocyst [5]. Two additional papers discussed blunt pancreatic trauma resulting in pancreatitis with or without formation of a secondary pancreatic pseudocyst [6,7].

## Case presentation

**Figure 1:**
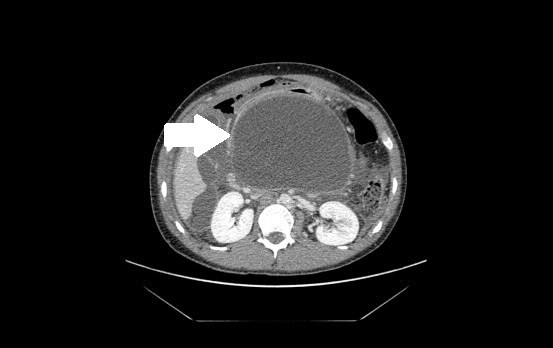
**CT scan demonstrating the large pancreatic pseudocyst and free gas within the abdomen**. This CT image, taken within a few hours of our patient's admission, shows the large 17 × 11 cm pseudocyst (indicated by the white arrow) and free gas within the abdomen.

A 23-year-old Asian male student presented as an emergency with an acute abdomen a week after minor trauma to his upper abdomen. The injury occurred during a night out when he was innocently punched in the abdomen by a friend. He experienced only moderate discomfort lasting a short time following the incident. His past medical history included coeliac disease and an admission four months previously with severe acute pancreatitis, believed to be due to alcohol binge drinking at the weekends. Ultrasound scanning at that time had shown no evidence of gallstone disease. Five days after the minor trauma, he became anorexic, lethargic and feverish and started vomiting bilious content. Seven days post-trauma, he presented to our emergency department with severe upper abdominal pain. On examination, our patient was found to be tachycardic, drowsy and in severe pain. Abdominal examination revealed a large firm epigastric mass. There was right upper quadrant tenderness with guarding. He was resuscitated and blood investigations revealed a high white blood cell count of 18.64 × 10^9^/L, a C-reactive protein level of 108 mg/L and an amylase level of 17 U/L. A chest radiograph demonstrated free air under his right hemidiaphragm. Our patient was initially managed with intravenous fluids, intravenous antibiotics, regular analgesia and antiemetics. A computed tomography (CT) scan of his thorax, abdomen and pelvis was obtained. This demonstrated a right pleural effusion, a large pancreatic pseudocyst measuring 17 × 11 cm (Figure 1) and thick free fluid within his abdomen with large pockets of air under his right hemidiaphragm. The site of perforation could not be determined from the CT images. A decision was made to proceed to an emergency laparotomy.

**Figure 2:**
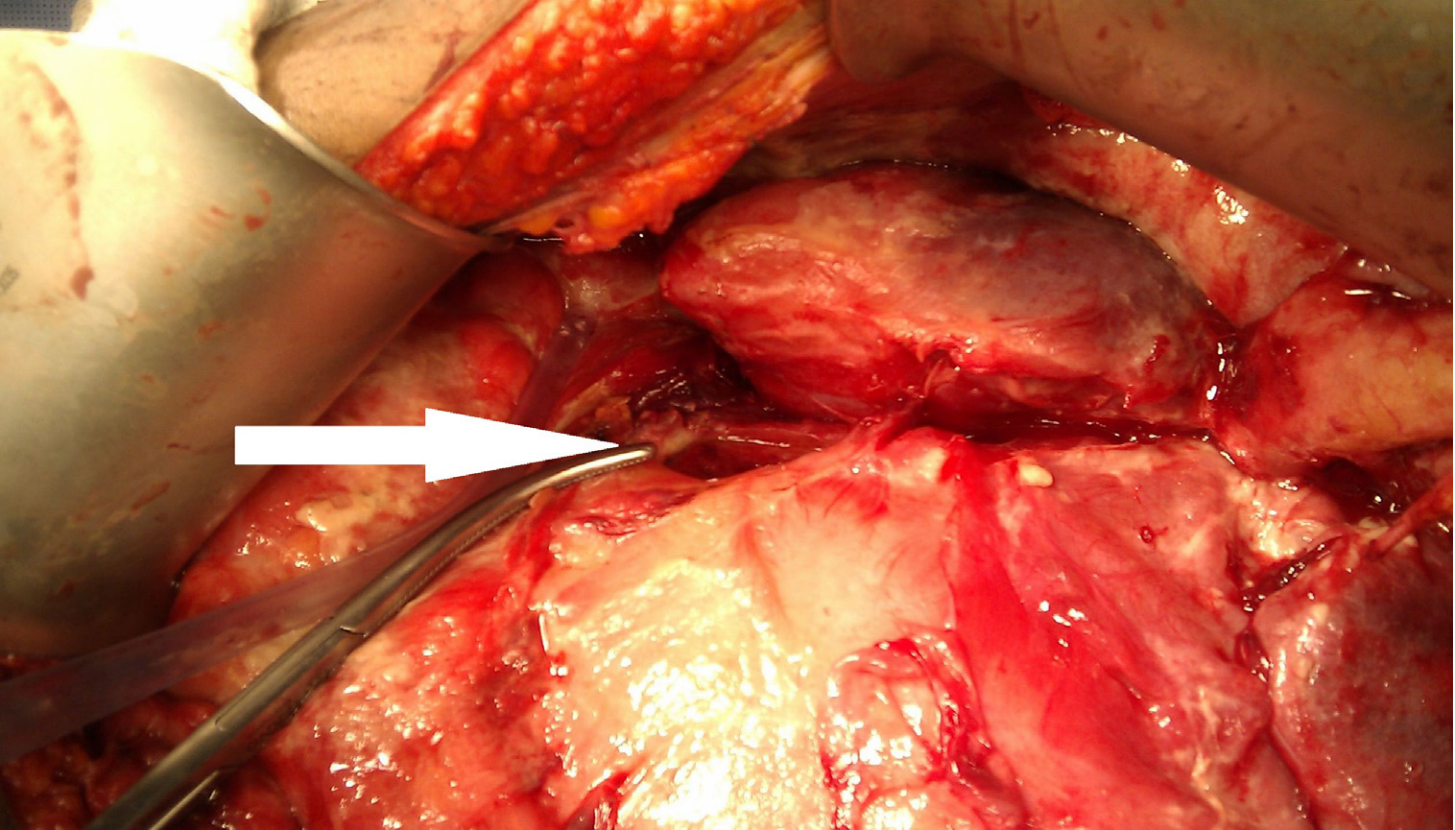
**The pancreatic pseudocyst**. The pseudocyst can be seen pushing the stomach and duodenum anteriorly at the time of surgery. The 5 cm transverse duodenal laceration is pointed out by the tip of the forceps (indicated by the white arrow).

**Figure 3:**
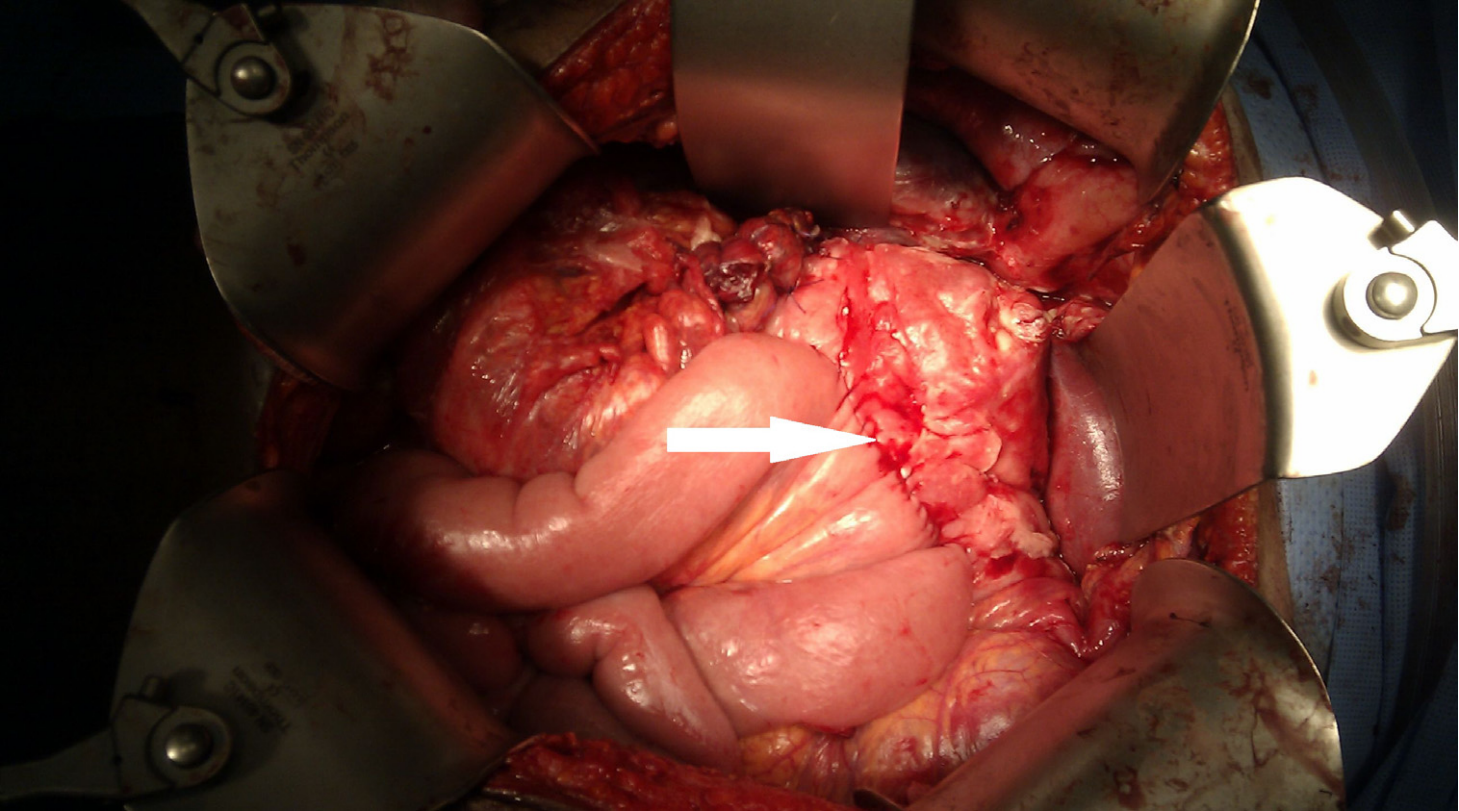
**The antecolic gastrojejunostomy fashioned to the anterior gastrostomy**. The antecolic gastrojejunostomy is indicated by the white arrow.

At laparotomy, free intraperitoneal air with loculated collections of bile and purulent fluids in the supra- and infracolic compartments were found. A large pancreatic pseudocyst was found, pushing the stomach and duodenum anteriorly with a 5 cm transverse laceration at the junction of the first and second part of duodenum (Figure 2). An anterior gastrostomy was made to drain the large pancreatic pseudocyst by a wide cystogastrostomy. The edges of the duodenal laceration were excised for histology and then closed transversely with interrupted absorbable sutures. An omental patch was sutured over the duodenal repair. The proximal jejunum was used to fashion an antecolic gastrojejunostomy to the anterior gastrostomy (Figure 3) to ensure drainage from his stomach. His abdominal cavity was lavaged with copious warm saline, a drain placed adjacent to the gastrojejunostomy and a drain by the duodenal repair and his abdomen closed. The drains were necessary due to the widespread peritoneal contaminations found during the laparotomy. He made an uneventful recovery and was discharged home on a proton pump inhibitor nine days later. Histology of the duodenal tissue showed no evidence of dysplasia or malignancy.

## Conclusion

Acute pancreatitis is a common condition presenting to general surgical and medical wards. Following acute pancreatitis, the incidence of a subsequent pancreatic pseudocyst ranges between 5% and 16% [8]. In cases of pancreatitis where alcohol is the cause, this figure rises to up to 78% [8]. Pancreatic pseudocysts do not always require treatment in an emergent setting [9] and are more often managed in an elective setting. The majority resolve spontaneously [9] and treatment is usually reserved for those which are symptomatic, larger than 6 cm and those which do not reduce in size during a six-week observation period [9,10]. This is the first reported case of a duodenal laceration following minor blunt abdominal trauma in the presence of a large pancreatic pseudocyst. Minor blunt abdominal trauma in a normal healthy adult would not be expected to result in any significant duodenal injury. We acknowledge that our patient may not have reported the truth regarding the severity of the trauma. However, this would be speculation and we based our premise on our patient's account of events. The absence of any external bruising to the abdominal wall on examination suggests that the blow was unlikely to have been severe, as we would expect this to occur with a severe blow. In this case, we postulated that the stretching of the duodenum over the large pancreatic pseudocyst had predisposed the duodenal wall to such an injury. The transverse linear laceration on the anterior duodenal wall at the junction of the first and second part of the duodenum would support the mechanism of the injury postulated. We postulate that an incomplete duodenal injury (such as a serosal tear) occurred following the blunt abdominal trauma, which then progressed to a complete laceration after a few days, when local tissue necrosis at the site of injury had taken place. This would result in the delayed clinical presentation of generalized peritonitis. We believe that patients with large pancreatic pseudocysts awaiting therapeutic interventions should be warned that minor blunt abdominal trauma could result in a life-threatening duodenal injury. It is also important that large pseudocysts are treated as early as possible to prevent possible complications.

## Consent

Written informed consent was obtained from the patient for publication of this case report and any accompanying images. A copy of the written consent is available for review by the Editor-in-Chief of this journal.

## Competing interests

The authors declare that they have no competing interests.

## Authors' contributions

VTM analyzed and interpreted the patient data regarding pancreatitis, pancreatic pseudocysts and gastrointestinal injuries following blunt trauma. VTM and MMS wrote the manuscript. MMS and LCT edited and amended the manuscript. LCT provided the photographs obtained at the time of surgery. All authors read and approved the final manuscript.
